# Educational Attainment and Smoking Status in a National Sample of American Adults; Evidence for the Blacks’ Diminished Return

**DOI:** 10.3390/ijerph15040763

**Published:** 2018-04-16

**Authors:** Shervin Assari, Ritesh Mistry

**Affiliations:** 1 Department of Psychiatry, University of Michigan, 4250 Plymouth Road, SPC 5763, Ann Arbor, MI 48109-2700, USA; 2 Center for Research on Ethnicity, Culture and Health, School of Public Health, University of Michigan, Ann Arbor, MI 48109-2029, USA; 3 Department of Health Behaviors and Health Education, School of Public Health, University of Michigan, Ann Arbor, MI 48109-2029, USA; riteshm@umich.edu

**Keywords:** population groups, race, Whites, Blacks, African-Americans, socioeconomics, education, smoking

## Abstract

Background: Although higher socioeconomic status (SES) indicators such as educational attainment are linked with health behaviors, the *Blacks’ Diminished Return* theory posits that the protective effects of SES are systemically smaller for Blacks than Whites. Aims: To explore the Black/White differences in the association between education and smoking. Methods: This cross-sectional study used the Health Information National Trends Survey (HINTS) 2017 (*n* = 3217). HINTS is a national survey of American adults. The current analysis included 2277 adults who were either Whites (*n* = 1868; 82%) or Blacks (*n* = 409; 18%). The independent variable was educational attainment, and the dependent variables were ever and current (past 30-day) smoking. Demographic factors (age and gender) were covariates. Race was the focal moderator. Results: In the pooled sample, higher educational attainment was associated with lower odds of ever and current smoking. Race interacted with the effects of higher educational attainment on current smoking, suggesting a stronger protective effect of higher education against current smoking for Whites than Blacks. Race did not interact with the effect of educational attainment on odds of ever smoking. Conclusions: In line with previous research in the United States, education is more strongly associated with health and health behaviors in Whites than Blacks. Smaller protective effects of education on health behaviors may be due to the existing racism across institutions such as the education system and labor market.

## 1. Introduction

### 1.1. Background

Smoking is the top cause of death in the US [1]. About 15.5% of all adults (17.5% of males and 13.5% of females) are current smokers [2], which is about 37.8 million people [3]. In the US, cigarette smoking causes more than 480,000 deaths annually, which is 1300 deaths every day [4]. Smokers die 10 years earlier on average compared to nonsmokers [5]. Total economic cost of smoking in US is more than $300 billion a year, including $170 billion in direct medical care [6] and more than $100 billion in lost productivity due to premature death [4].

In the US, socioeconomic status (SES) is a strong determinant of smoking prevalence, smoking severity, smoking duration, and acquiring health conditions due to smoking [7]. People living above the poverty level and people who have high educational attainment have lower rates of cigarette smoking than the general population [8,9]. Smoking prevalence is highest among people with only a GED certificate (40%), compared to any other SES group [9]. People with high SES also tend to smoke cigarettes less heavily. People with a family income of three times the poverty rate smoke cigarettes for a duration of less than half that of people in poverty [10]. People with at least a bachelor’s degree smoke cigarettes for a duration of half that of people with a high school education [10]. Although SES does not impact the likelihood of making quit attempts, high SES individuals are more likely to quit smoking cigarettes than low SES individuals [8]. Some of the additional burden of smoking in low SES populations is due to the higher density of tobacco retailers [11] and advertising campaigns of the tobacco industry [8] in low-income neighborhoods [11]. Tobacco companies have historically targeted low SES populations through the distribution of discount coupons, point-of-sale discounts, direct-mail coupons, and the development of brands that appeal to them [12].

According to the Blacks’ Diminished Return theory [13,14], healthful effects of higher SES are smaller for Blacks compared to Whites. There are unequal gains when the distribution of socioeconomic resources is the same across racial groups, which explains the persistent racial health gaps despite enormous investments in the US to achieve health equity [14]. Social and economic resources such as education [15,16], employment [17,18], neighborhood quality [19], and social contacts [20] all have stronger protective effects in Whites than Blacks. It is still unknown, however, whether the Blacks’ Diminished Return theory is valid for smoking, despite smoking being the number one cause of death.

The smaller health gains for Blacks compared to Whites have been attributed to institutional racism. How SES affects health is dependent on race, which shapes how individuals can access resources, navigate institutions, and have personal autonomy over their lives [13,14]. As Blacks are more likely to be treated unfairly and unjustly than Whites, there is a perpetual disadvantage compared to Whites in benefitting from the opportunity structures. In addition, pervasive racism and discrimination increases the psychosocial stress of upward social mobility for non-Whites. For example, residential and job segregation, the lack of a universal health care access, and inadequate social safety nets in the US increase the challenges of upward social mobility experienced by minority groups [13]. Enormous racial gaps in wealth accumulation and SES exist across the life course [13,14,21,22].

A considerable body of research has recently shown that the health gain of SES indicators such as education is smaller for Blacks than Whites. In a 15-year longitudinal study of a birth cohort, SES at birth had protective effects against future obesity among Whites but not Blacks [23]. Similar results are shown for the effects of education on alcohol use [15], physical activity, diet [24], breastfeeding, hunger [24], sleep quality [25], self-rated health [26,27], and depression [28,29,30]. We are, however, not aware of any study about Blacks’ Diminished Return of education on smoking.

### 1.2. Aim

To extend the current knowledge on the Blacks’ Diminished Return theory [13,14], and in continuation of previous work on the multiplicative effects of race and education on health [28,29], this study investigated whether education, a central indicator of SES, had smaller effects on smoking for Black than White adults in the US. We hypothesized a weaker association of education and smoking for Blacks than Whites.

## 2. Methods

### 2.1. Design and Setting

The current study used the most recent version of HINTS (also called HINTS 5, Cycle 1), which was conducted between January and May 2017. HINTS is a nationally-representative survey that has been periodically administered by the National Cancer Institute (NCI) since 2003. The HINTS target population is non-institutionalized American adults (age ≥ 18) who reside in the US. The primary purpose of the HINTS is to provide a comprehensive assessment of the American adults’ access to and use of information about cancer [31,32,33].

### 2.2. Ethics

All participants provided informed consent. HINTS 5 was approved by the Westat’s Institutional Review Board (B03-00004038-R1). HINTS was deemed exempt from IRB review by the NIH Office of Human Subjects.

### 2.3. Sampling

The HINTS 5, Cycle 1 sampling strategy consisted of a two-stage design. The first stage was a stratified sample of addresses derived from all residential addresses. The second stage included the selection of one adult from each sampled household. The list of addresses was provided by the Marketing Systems Group (MSG). All non-vacant residential addresses in the US were eligible for sampling. The sampling frame of addresses was grouped into two sampling strata: (1) areas with a high concentration of minorities; and (2) areas with a low concentration of minorities. An equal-probability sample of addresses was selected from each sampling stratum [34].

### 2.4. Surveys

The survey was conducted exclusively by mail. Two toll-free telephone numbers were provided to respondents: one was used for English calls and one was used for Spanish calls [34].

### 2.5. Measures

*Demographic variables*. Age and gender were collected at baseline (in 1995). Age was operationalized as a continuous measure. Gender was operationalized as a dichotomous variable (male was the reference group).

*Educational attainment*. Education was the main SES indicator in our study. Educational level was reported as (1) less than high school, (2) high school graduate, (3) some college, (4) bachelor’s degree, and (5) post-baccalaureate degree. Education was operationalized as a continuous measure, with higher scores reflecting more education.

*Smoking Status*. Smoking status was measured using the following items: (1) “Have you smoked at least 100 cigarettes in your entire life (yes/no)?”, and (2) “Do you currently smoke?” Response options to both questions were yes/no. Using these items, we calculated two variables: ever smoker and current smoker [35,36,37,38]. These items have been used in National Health and Nutrition Examination Survey (NHANES) and several other major national behavioral surveillance systems [39]. Self-reported smoking has shown strong concurrent, criterion, and divergent validity [40]. National surveys of the general population show no significant difference between national estimates of smoking prevalence based on self-reported versus urinary cotinine concentrations, indicating that self-reported data on smoking status provide a valid estimate of the prevalence of smoking [41]. As in the NHANES, ever smokers were defined as individuals who had smoked 100 or more cigarettes in their entire life. Current smokers were defined as ever smokers who reported smoking at the time of study [42].

*Moderator*. Self-identified race, conceptualized as a social construct, was measured [43]. Race was operationalized as a dichotomous variable (Whites = 0 [reference group], Blacks = 1). We only included Blacks and Whites in the study because the aim was to test Black-White differences.

### 2.6. Statistical Analysis

*Data Analysis*. We used Stata 13.0 (Stata Corp., College Station, TX, USA) for our data analysis. We applied sampling weights available in the HINTS public us files to account for strata, clustering, and non-response. Jackknife standard errors were calculated. For univariate statistics, we described weighted mean and proportions (frequencies), along with their standard errors. We used Stata’s *svy* commands with the *subpop* option for all our analyses. For bivariate analysis, we used an independent sample *t* test and Pearson Chi square tests to compare Blacks and Whites. For multivariable analysis, we estimated four logistic regression models. In these logistic regression models, we used education as the independent variable, current or ever smoking as the dependent variables, demographics (age and gender) as covariates, and race as the focal moderator. First, two logistic regressions were estimated in the pooled sample. The first model did not include race by education interaction. We then ran a model with the race by education interaction term. Subsequently, we performed race-specific logistic regressions (*Model 3* for Whites and *Model 4* for Blacks). Adjusted odds ratio (OR), 95% Confidence Intervals (CI), and *p* values were reported. Age and education were operationalized as a continuous measure. As such, the ORs for age were indicative of differences in the odds ratio of smoking comparing a one unit increase in age and education. A *p* of value less than 0.05 was considered statistically significant.

## 3. Results

### 3.1. Descriptive Statistics

This study included 2277 adults who were either Whites (*n* = 1868, 82%) or Blacks (*n* = 409; 18%). Table 1 provides descriptive statistics of the study variables in the overall sample and by race. Blacks had lower education than Whites. Blacks reported less ever smoking than Whites; however, current smoking was not different between Blacks and Whites (Table 1).

### 3.2. Multivariable Models for Current Smoking Status

Table 2 presents the results of four logistic regression models with education as the independent variable and current smoking as the dependent variable. *Models 1* and *2* were estimated in the pooled sample. *Model 1* only included the main effects of education and race. *Model 2* also included an interaction term between race and education. Based on *Model 1*, high education was associated with lower odds of current smoking above and beyond all covariates. *Model 2* showed a significant interaction between race and education on current smoking, suggesting that the protective effects of education on current smoking are larger for Whites than Blacks. *Model 3* was estimated in Whites and *Model 4* in Blacks. *Model 3* showed that high education was associated with lower odds of current smoking for Whites. *Model 4* showed a marginally protective effect of education on current smoking for Blacks (Table 2).

### 3.3. Multivariable Models for Ever Smoking Status

Table 3 presents the results of four logistic regression models with education as the independent variable and ever smoking status as the dependent variable. Aside from a different outcome, the models were setup similarly to those shown in Table 2. Based on *Model 1*, high education was associated with lower odds of ever smoking above and beyond all covariates. *Model 2* did not show any interaction between race and education on ever smoking. *Model 3* showed that high education was associated with lower odds of ever smoking for Whites. *Model 4* also showed a protective effect of education on ever smoking for Blacks (Table 3).

## 4. Discussion

Using nationally representative data, this study explored Black-White differences in the protective effects of educational attainment on smoking status. The current study found a stronger inverse association between education and current smoking status among Whites than Blacks.

Our finding about the main effect of education against smoking is consistent with previous research, which has shown that high SES is protective against smoking in the US [8,9]. Education [8] and income [9] are both protective against the prevalence, severity, duration, and consequences of smoking [7]. Smoking prevalence is highest among people having only a General Equivalency Diploma (GED) certificate (40%), compared to any other SES group [9]. This study, we believe, is the first to test the Blacks’ Diminished Return theory when smoking is a health outcome.

The racial differences in the effects of education on smoking status that we observed are in line with stronger protective effects in Whites than Blacks for several other risk factors [44,45,46,47,48,49]. Studies on correlates of smoking in Whites and Blacks have found many more significant associations for Whites than Blacks [44]. In one study, maternal religiosity and scholastic attitudes were protective against smoking in Whites but not Blacks [44]. The protective effects of having peers [47,49] and parents [48] who do not smoke are stronger in Whites than Blacks. Thus, it is not just education but many other protective factors that have smaller effects for Blacks.

Our findings are important given that smoking is one of the most salient behavioral risk factors for morbidity and mortality in the US and for low SES groups and racial minorities such as Blacks [50]. The findings help explain why the consequences of smoking and other substances are disproportionately worse for Blacks compared to Whites [51,52]. The Blacks “telescoping effect” suggests that on average Blacks transition more rapidly from gateway substances such as smoking and alcohol to heavier substances and illicit drugs [53,54]. At the same time, Blacks may have lower success with quitting cigarettes [55].

The observed racial inequalities in the protective effects of education on smoking may be due to inequalities in the education system, labor market, and other institutions. Lower education quality due to the scarcity of educational resources within majority Black areas may be responsible for the observed differential effect of education across races [56]. The educational system also discriminates against Blacks [57], which reduces the gains that follow education. The magnitude of the education effect on health is conditional upon how education translates into income and wealth, which itself depends on race [58,59,60,61]. Despite anti-discrimination regulations, the labor market continues to provide less employment opportunities for non-White racial groups than Whites [62]. Labor market preferences favor Whites, thus reducing the benefits of education for minority groups including Blacks [63]. For example, the labor market discriminates against Blacks in hiring and wages [64,65,66,67,68]. Due to residential segregation, Blacks may have difficulty with commuting to work and competing for high paying jobs [69]. Applicants with Black names are less likely to be selected for job interviews [70,71,72]. In 2006, among men with a master’s degree, Blacks earned $27,000 less than Whites [16,73]. Dual market theory suggests that occupations are roughly divided into two categories: primary jobs that have high wages, good working conditions, and opportunities for advancement; and secondary jobs with minimum wages, poor working conditions, stressful and unstable conditions, and minimal opportunity for growth and promotion [74]. At each level of education, Blacks are more likely to work in secondary jobs [75]. At each level of education, Blacks are exposed to more stress that hinders their health gain. SES resources have smaller protective effects in Blacks than Whites in the presence of several other risk factors [76,77,78,79,80,81]. Highly educated Blacks do not enjoy equal access to the opportunity structure compared to their White counterparts [82,83]. All these mechanisms result in a smaller health gain from education for Blacks compared to Whites.

The differential effect of SES on smoking by race may be due in part to the policies and practices of the tobacco industry and access to smoking cessation services that disproportionately affect racial minorities. At least some of this pattern may be due to factors such as predatory marketing [84], flavoring [85], and the density of tobacco retailers [86,87]. Blacks may be also more sensitive to tobacco industry marketing than Whites [88]. There is evidence of predatory marketing in poor and minority neighborhoods [84]. There is flavor branding according to race (mentholated cigarettes targeting Blacks) [85]. Although the Food and Drug Administration (FDA) has banned tobacco flavoring, menthol, which disproportionately affects Blacks, is still an exception [85]. There is poor access to cessation services in urban areas where most Blacks live [89]. Even with access to cessation services, the impact of such programs is smaller for Blacks than Whites [89].

The current study extends previous evidence by examining smoking as an outcome. The finding that there are smaller protective effects of educational attainment on smoking outcomes among Blacks compared to Whites is in line with the results of other studies on racial differences in the effects of education on other behaviors and health outcomes. Smaller effects of education on alcohol consumption [15], diet [74], and depression [28,29,30] are shown for Blacks compared to Whites. Other studies have shown larger protective effects of education on mortality for Whites than Blacks [16,18,65,90,91]. Non-additive effects of race and SES on health support the views of Navarro [92], Williams [93], and Mehta [94], who have argued that race and SES interact in terms of health. Health disparities should be seen as a consequence of complex nonlinear inter-related interactions between race and SES resources that disfavor minorities. Blacks experience less of the protective influence of higher SES than Whites. That is, socioeconomic resources better serve the majority then the minority group.

### 4.1. Implications

The results have several implications. First, smoking is the main cause of mortality in the US, and worldwide, and in minority populations such as Blacks [1]. The unequal effect of education on smoking favoring whites may help explain some of the racial differences in mortality due to smoking in the US. Second, the results contribute to better understanding racial differences in health consequences of smoking [95]. Although smoking rates are somewhat similar for Blacks and Whites [95], and SES is protective against smoking [95], race and class interact to shape smoking-related health disparities. Third, the results about the interactions of SES and race on smoking may help design programs for tobacco use prevention. For example, programs that serve participants of a similar SES should provide more program dose and other strategies to Black participants because they may experience less of the protective effects from SES resources than their White counterparts. While overall, low SES individuals need more investment for smoking prevention and cessation programs compared to high SES individuals [8,9], this distinction seems to be more relevant to Whites than Blacks. High SES Blacks need more exposure to tobacco prevention and cessation programs than high SES Whites. Closing the gap between White and Black health is likely not possible by eliminating the racial gap in SES alone.

### 4.2. Limitations

This study is not free from limitations. First, with a cross-sectional design, our study does not allow any causal inferences. Longitudinal studies are needed to observe how baseline SES impacts change in smoking behaviors over time. Thus, there is a need to replicate these findings using longitudinal data. Second, we had two simple measures of smoking. But, these measures are widely accepted and used in many behavioral risk factors surveillance systems in the US and worldwide [39,42]. For examples, our definition of current and ever smoking was similar to that of the National Health and Nutrition Examination Survey (NHANES) [39,42]. Future research should use more comprehensive measures of smoking behavior that focus on daily smoking, such as number of cigarettes smoked per day and quitting. Third, potential underlying mechanisms behind the observed differential effects were not investigated. Family structure, childhood SES, wealth, employment, and psychosocial stress may be some explanatory factors. Lastly, due to the lower sample size of Blacks than Whites, this study had a lower statistical power in Blacks than Whites. Despite the above limitations, this study extends the existing literature on the Blacks’ Diminished Return theory and the interactive effects of race and SES on smoking. The nationally representative sample is an advantage of this study. The prevalence of smoking outcomes in our sample of Whites and Blacks was comparable to reports by CDC [7,9], which is indicative of the robust sampling methodology for the survey data used in this study.

## 5. Conclusions

To conclude, race modifies the protective effects of education on health outcomes, and smoking is not an exception. That is, in America, education constantly generates a greater benefit for the majority than for the minority group. There is a need for policies and programs that reduce Blacks’ diminished returns of education. Policies should go beyond increasing minorities’ access to SES resources, and identify ways to increase Blacks’ capacity to use them. There is a need to reduce discrimination across levels such as the labor market, education system, and correctional setting. There is a need to reduce structural and institutional racism in the US. Without such changes, merely eliminating the racial gap in access to SES resources will not be enough to eliminate the racial gap in health.

## Figures and Tables

**Table 1:** Descriptive statistics in the overall sample and by race.

	All (*n* = 2277)	Whites (*n* = 1868)	Blacks (*n* = 409)
	Mean or % (SE)	Mean or % (SE)	Mean or % (SE)
Age	48.80 (0.34)	50.10 (0.46)	47.72 (1.22)
Education *	3.12 (0.02)	3.17 (0.02)	3.08 (0.10)
Gender			
Female	50.63 (0.00)	50.84 (0.00)	60.86 (0.04)
Male	49.37 (0.00)	49.16 (0.00)	39.14 (0.04)
Education			
Less than High School	8.37 (0.01)	5.54 (0.01)	13.69 (0.03)
High School Graduate	22.67 (0.01)	20.16 (0.01)	24.01 (0.03)
Some College	32.98 (0.01)	41.03 (0.01)	19.36 (0.03)
Bachelor’s Degree	22.38 (0.01)	20.37 (0.01)	26.04 (0.04)
Post-Baccalaureate Degree	13.60 (0.01)	12.91 (0.01)	16.91 (0.04)
Smoking			
Current	16 (0.01)	16 (0.02)	16 (0.02)
Ever *	38 (0.01)	45 (0.02)	29 (0.04)
Former *	23 (0.01)	28 (0.01)	14 (0.03)
Never *	62 (0.01)	55 (0.02)	71 (0.04)

Weighted means and percent values along with standard errors are provided. * *p* < 0.05 for comparison of Blacks and Whites.

**Table 2:** Summary of logistic regression on current smoking models in the overall sample.

	All (*n* = 2277)				Whites (*n* = 1868)		Blacks (*n* = 409)	
	OR	95% CI	OR	95% CI	OR	95% CI	OR	95% CI
	*Model 1*		*Model 2*		*Model 3*		*Model 4*	
Race (Blacks)	0.87	0.50–1.52	# 0.40	0.16–1.05				
Gender (Male)	1.25	0.76–2.05	1.26	0.77–2.06	1.13	0.64–1.99	# 2.61	0.95–7.12
Age	** 0.98	0.97–0.99	** 0.98	0.97–0.99	** 0.98	0.96–0.99	1.01	0.98–1.04
Education (1–5)	*** 0.59	0.49–0.71	*** 0.56	0.45–0.69	*** 0.55	0.45–0.69	# 0.79	0.60–1.04
Race * Education	-	-	* 1.33	1.00–1.78				
Intercept	2.24	0.68–7.42	2.62	0.77–8.93	# 3.32	0.91–12.07	# 0.16	0.02–1.28

# *p* < 0.1, * *p* < 0.5, ** *p* < 0.01, *** *p* < 0.001.

**Table 3:** Summary of logistic regression models on ever smoking in the overall sample.

	All (*n* = 2277)				Whites (*n* = 1868)		Blacks (*n* = 409)	
	OR	95% CI	OR	95% CI	OR	95% CI	OR	95% CI
	*Model 1*		*Model 2*		*Model 3*		*Model 4*	
Race (Blacks)	** 0.53	0.34–0.85	0.74	0.24–2.27				
Gender (Male)	* 1.44	1.06–1.96	* 1.44	1.06–1.95	# 1.36	0.99–1.88	# 2.25	0.96–5.29
Age	* 1.01	1.00–1.03	* 1.01	1.00–1.03	* 1.01	1.00–1.03	1.02	0.99–1.06
Education (1–5)	*** 0.73	0.64–0.83	*** 0.74	0.63–0.87	*** 0.74	0.63–0.87	** 0.67	0.51–0.88
Race * Education			0.89	0.65–1.23				
Intercept	0.88	0.36–2.18	0.84	0.33–2.14	0.90	0.34–2.36	0.30	0.04–2.10

# *p* < 0.1, * *p* < 0.5, ** *p* < 0.01, *** *p* < 0.001.
