# Erratum: Assari, S.; Mistry, R. Educational Attainment and Smoking Status in a National Sample of American Adults; Evidence for the Blacks’ Diminished Return. *Int. J. Environ. Res. Public Health* 2018, *15*, 763

**DOI:** 10.3390/ijerph15102084

**Published:** 2018-09-21

**Authors:** Shervin Assari, Ritesh Mistry

**Affiliations:** 1 Department of Psychiatry, University of Michigan, 4250 Plymouth Road, SPC 5763, Ann Arbor, MI 48109-2700, USA; 2 Center for Research on Ethnicity, Culture and Health, School of Public Health, University of Michigan, Ann Arbor, MI 48109-2029, USA; 3 Department of Health Behaviors and Health Education, School of Public Health, University of Michigan, Ann Arbor, MI 48109-2029, USA; riteshm@umich.edu

Due to an error during production, a citation of the published paper [1] was incorrect. The corrected citation is provided below.

**Incorrect reference:**

Mackenbach, J.D.; Tiemeier, H.; Ende, J.; van der Nijs, I.M.T.; Jaddoe, V.W.V.; Hofman, A.; Jansen, P.W. Relation of emotional and behavioral problems with body mass index in preschool children: The generation R study. *J. Dev. Behav. Pediatr.* **2012**, *33*, 641–648, doi:10.1097/DBP.0b013e31826419b8.

**Correct reference:**

Mackenbach, J.P.; Stirbu, I.; Roskam, A.J.; Schaap, M.M.; Menvielle, G.; Leinsalu, M.; Kunst, A.E. European Union Working Group on Socioeconomic Inequalities in Health. Socioeconomic inequalities in health in 22 European countries. *N. Engl. J. Med.* **2008**, *358*, 2468–2481, doi:10.1056/NEJMsa0707519.

This change does not modify the results, discussion, or the conclusions. The authors would like to apologize for any inconvenience caused to the readers by this error. The article will be updated and the original will remain on the article webpage.
